# Associations between gestational weight gain and pregnancy outcomes using WHO, Asian, and Indonesian BMI classifications: a prospective longitudinal study in Parepare, Indonesia

**DOI:** 10.1186/s12889-026-26818-2

**Published:** 2026-03-14

**Authors:** Sabaria Manti Battung, A. Razak Thaha, Eline M. van der Beek, Henk Groen

**Affiliations:** 1 Department of Pediatrics, University of Groningen, University Medical Center Groningen, Groningen, The Netherlands; 2 Department of Nutrition, Faculty of Public Health, Hasanuddin University, Makassar, Indonesia; 3 School of Public Health, University of Muhammadiyah Jakarta, Jakarta, Indonesia; 4 Nestlé Institute of Health Sciences, Nestlé Research, Societé du Produits Nestlé Lausanne, Lausanne, Switzerland; 5 Department of Epidemiology, University of Groningen, University Medical Center Groningen, Groningen, The Netherlands

**Keywords:** Body mass index, Gestational weight gain, Asian, Indonesian, Low birth weight, Small for gestational age, Preterm birth, Pregnancy outcomes

## Abstract

**Background:**

Current recommendations for adequate gestational weight gain (GWG) are based on body mass index (BMI) classes proposed by the WHO. However, for Asian populations alternative BMI classifications have been suggested. This study aims to explore the associations between GWG and pregnancy outcomes using WHO, Asian and Indonesian BMI cutoffs.

**Methods:**

A prospective longitudinal study was conducted in the Parepare district, Indonesia. Of 1216 pregnant women enrolled, 953 mother-child dyads had complete newborn data. BMI was categorized into: underweight, normal weight, overweight, and obesity according to WHO, Asian and Indonesian BMI cutoffs. IOM criteria were used to determine GWG adequacy. Adverse pregnancy outcomes were: small for gestational age, preterm birth and low birth weight. For all three BMI classifications, logistic regression analyses were performed to examine the association between GWG and pregnancy outcomes. Additionally, we conducted a goodness of fit test to compare the model performance for each BMI classification method.

**Results:**

The prevalence of underweight was 11.4% regardless of BMI classification. The prevalence of overweight was 22.2%, 33.3%, and 11.8%, whereas the prevalence of obesity was 6.8%, 14.4%, and 17.3% using the WHO, Asian and Indonesian cutoffs, respectively. Across all BMI classifications, a considerable proportion of women who were classified as underweight (> 70%) or normal weight (> 69%) exhibited inadequate GWG. The Asian BMI classification had the highest proportion of excessive GWG (15.2%).

**Conclusion:**

Gestational weight gain classes based on Asian BMI cutoffs corresponded better with adverse pregnancy outcomes than WHO or Indonesian BMI cutoffs. We hypothesize that implementing the Asian BMI classification may better identify risks for adverse pregnancy outcomes, enabling more accurate and timely interventions targeting (pre)pregnancy to improve outcomes for mother and child.

**Supplementary Information:**

The online version contains supplementary material available at https://doi.org/10.1186/s12889-026-26818-2.

## Introduction

Maternal nutritional status in the perinatal period plays a crucial role in determining maternal and child health. Nutritional status is determined based on pre-pregnancy body mass index (BMI) and affects gestational weight gain (GWG), and the risk of inadequate and excessive GWG have been shown to be associated with various adverse pregnancy outcomes. Maternal undernutrition (BMI < 18.5 kg/m^2^) increases the risk of low GWG and pregnancy outcomes like preterm birth and small for gestational age, whereas maternal obesity is associated with the risk of macrosomia or large birth weight for gestational age as well as the risk of gestational diabetes mellitus, preeclampsia, increased delivery via caesarean section, postpartum weight retention and offspring obesity [1–7]. Therefore, using appropriate pre-pregnancy BMI cutoffs and guiding adequate GWG during pregnancy is essential for minimizing the risk of adverse outcomes for both mother and child.

There is currently, however, no global consensus regarding population-specific optimal weight gain during pregnancy [8]. Most GWG charts have been derived from high-income countries and/or lack coverage for the full range of pre-pregnancy BMI [9–11]. The Institute of Medicine (IOM) GWG recommendation is the most commonly used, but may not always be suitable as it is based on observational data from the US population [12]. More specifically, these recommendations may not be suitable for Asian women who tend to be shorter and thinner than the average American woman. Additionally, Asian people have a higher body fat percentage at comparable BMI, and those with a BMI < 24.5 kg/m^2^ face a higher relative risk of developing cardiovascular disease and diabetes type II [13, 14].

Evidence-based guidelines for appropriate GWG in Asian populations are currently lacking. Some studies suggested that optimal GWG in Asian pregnant women may be lower than the IOM recommendations, particularly for women with overweight and obesity [15, 16]. The 2004 WHO expert consultation proposed alternative BMI cutoffs for Asians with lower values to define overweight and obesity compared to the WHO standard [13]. Most Asian countries have either adopted these thresholds or slightly modified them based on their population data. In addition, there is an ongoing WHO effort to develop global GWG standards that can be applied to all pre-pregnancy BMI levels across diverse geographies [17].

While most Asian countries use Asian BMI cutoffs, Indonesia continues to rely on the standard WHO BMI cutoffs for assessing GWG adequacy. This approach may lead to an underestimation of the prevalence of overweight and obese individuals. The prevalence of overweight/obesity in Indonesian women aged > 18y shows a substantial increase from 32,9% [18] to 44.4% in 2018 [19]. In parallel, the prevalence of type II diabetes has shown a clear upward trend among adults. It is projected to further increase from 9.19% in 2020 (18.7 million cases) to 16.09% in 2045 (40.7 million cases) [20]. These trends suggest it is likely that more women will enter pregnancy being overweight or obese which may increase the risk of developing gestational diabetes mellitus (GDM). A small study conducted in Indonesia involving 195 pregnant women revealed that approximately 10.2% were underweight and 43.1% were overweight/obese based on standard WHO BMI cutoffs. Also, 53.3% experienced inadequate GWG and 12.3% had excessive GWG [21].

There is ongoing debate about whether standard WHO BMI cutoffs should be applied to Asians, especially given the high prevalence of overweight/obesity and type II diabetes in the region. Very few studies have focused on BMI classifications and GWG in Indonesia, and none have compared the impact of using WHO, Asian and Indonesian BMI cutoffs. Therefore, in this study we performed an explanatory analysis to investigate associations between gestational weight gain and pregnancy outcomes using Asian, WHO and Indonesian BMI classifications.

## Methods

### Study design and participants

**Fig. 1:**
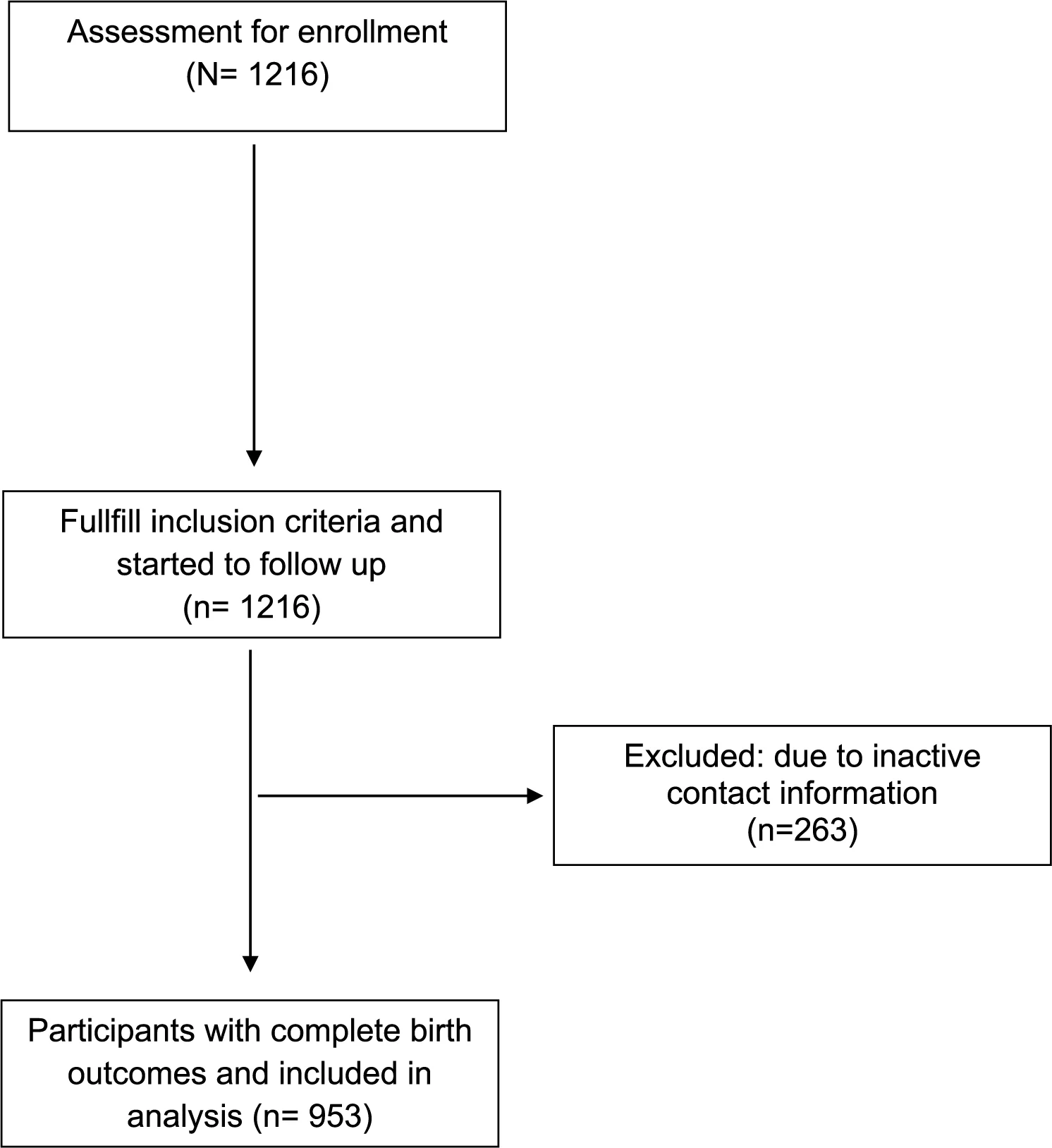
Strobe flow chart

This study used data from an observational longitudinal study of multiple micronutrient supplementation conducted in Parepare, Indonesia collected from August 2021 to October 2022 in eight community health centers [22]. The sample size was calculated based on the assumed difference in average number of MMS tablets consumed between mothers delivering either low birth weight or normal weight infants. The study assumed a 5% prevalence of low birth weight and an effect size ranging from 0.4 to 0.5 for MMS tablet consumption. To achieve 80% power to detect this effect size with a two-sided alpha level of 5%, indicating reduced MMS exposure in low birth weight infants, a minimum of 1260 mother-infant pairs was required, consisting of 60 low birth weight infants and 1200 normal weight infants. The inclusion criteria were (1) women who were willing to consume MMS; (2). Women who had started MMS before inclusion were also allowed to participate if the start of MMS use was documented in the MCHB. The former was determined by asking these women during the first ANC whether they wanted to consume the MMS and participate in this study, whereas the latter was determined by checking the remaining tablets in their current prescription bottle to check intake up to the recruitment visit. (3) Participating women were required to be planning to stay in the town during pregnancy, and to have a MCHB. The exclusion criteria were: (1) women who did not have a complete history of MMS consumption; (2) women who stay temporarily in the study location. A total of 1216 pregnant women were enrolled in the study and monitored from enrollment through delivery. However, data on newborn weight was unavailable for 263 participants due to loss to follow-up. As a result, 953 mother-dyads data were included in the present analysis (Fig. 1). Written informed consent was obtained from all participants.

### Data collection and measurement

A survey-based questionnaire was used to collect the data (Supplementary File 1), which was subsequently entered into a secure electronic database using REDCap (Research Electronic Data Capture, projectredcap.org). Two trained midwives were assigned for data collection at each community health center.

Data on pre-pregnancy weight in kilograms (kg) was self-reported, whereas height was measured using a wall-mounted measuring tape microtoise in the first ANC visit. Weight gain was measured using Seca 877 scale. Women were weighed in light clothes and without shoes. Pre-pregnancy BMI was calculated by dividing pre-pregnancy weight by height squared. BMI then was categorized into: underweight, normal weight, overweight, and obesity according to the WHO [23] standard or Asian and Indonesian general population BMI cutoffs [19]. The latter definition is further referred to as Indonesian cutoffs. GWG was calculated by subtracting the pre-pregnancy weight from the measured in the third trimester. The IOM provides different recommendations for GWG ranges for pregnancy according to pre-pregnancy BMI [12] (Table 1). Women were classified as experiencing inadequate, adequate or excessive GWG during pregnancy based on WHO, Asian and Indonesian BMI classifications.

**Table 1:** Pre-pregnancy body mass index and recommendations for gestational weight gain during pregnancy

Pre-pregnancy BMI	WHO classification	ASIAN classification	Indonesian classification	IOM recommended GWG (kg)*
Underweight	< 18.50	< 18.50	< 18.50	12.5–18.0
Normal weight	18.5–24.9	18.5–22.9	18.5 – <25.0	11.5–16.0
Overweight	25.0–29.9	23.0–27.4	25.0 – < 27.0	7–11.5
Obese	≥ 30.0	≥ 27.5	≥ 27.0	5.0–9.0

^*^based on WHO classification

Data on newborns, including sex, birth weight, birth length, head circumference, delivery method, and gestational age at delivery, were obtained from MCHB filled by midwives shortly after the babies were born. All participants delivered their babies either in hospitals or community healthcare facilities. To collect newborn data, we asked participants to photograph the relevant pages containing newborn data in their MCHB. These images were then shared with us via WhatsApp and integrated with the existing maternal characteristics data, linking each newborn’s data to their respective mother’s record.

The adverse pregnancy outcomes were defined using: (1) small for gestational age (SGA); as < 10th percentile of birthweight for sex and gestational age using INTERGROWTH-21st Project that can be used across multiethnic populations [24] (2) preterm birth (PTB); e.g. spontaneous birth < 37 weeks gestation; (3) low birth weight; as weight (LBW) at birth < 2500 gram; (4) large for gestational age (LGA); as > 90th percentile of birthweight for sex and gestational age; (5) macrosomia; a birth weight > 4000 gram, and finally a composite adverse outcome defined as the presence of one or more of the following outcomes: SGA, PTB and LBW.

### Statistical analysis

The data were analyzed using SPPS Statistics for Windows version 28.0. Characteristics of participants were summarized using mean, SD and proportions. The distribution of GWG by pre-pregnancy BMI was summarized by proportion. A linear-by-linear analysis using crosstabulation was conducted to examine a linear trend between GWG categories and pregnancy outcomes. Furthermore, we performed logistic regression analysis to assess the association between GWG categories and pregnancy outcomes with adequate GWG as the reference. The results were reported as odds ratio with 95% CI and statistical significance was set at *p* < 0.05. We calculated the Akaike Information Criterion (AIC) [25] for the logistic regression models examining the association between GWG and pregnancy outcomes using WHO, Asian and Indonesian cutoffs to determine which model performed better.

### Ethics

Approval for the study was obtained from the University of Hasanuddin’s Ethical Committee (7267/UN4.14.1/TP.01.02/2021) before the start of inclusion.

## Result

Of 1216 pregnant women recruited, 953 had data on newborns and were included in this analysis (Table 2). The mean maternal age at inclusion was 28.0 ± 5.7 years, pre-pregnancy weight was 54.2 ± 10.4 kg, height was 152.7 ± 5.0 cm, and GWG was 8.9 ± 4.5 kg. For the newborns, the mean birth weight was 3077 ± 406.3 g and the birth length was 48.2 ± 1.7 cm.

**Table 2:** Characteristics of participants

Characteristic	Mean ± SD or *n* (%) (*n* = 953)
*Sociodemographic*	
Age at inclusion	28.0 ± 5.7
Education at university or diploma level	689 (72.3)
Occupation/has job	188 (19.7)
Owning health insurance	910 (95.5)
Indoor passive smoking	559 (58.7)
*Anthropometric indicators*	
Pre-pregnancy Weight (kg)	54.2 ± 10.4
Height (cm)	152.7 ± 5.0
MUAC at 3rd trimester (cm)	27.6 ± 3.2
*Obstetric history*	
Primiparous	303 (31.8)
*Inter pregnancy interval*	
* First pregnancy*	397 (32.6)
≤ 2y	350 (28.8)
* > 2y*	469 (38.6)
Abortion history	148 (15.5)
HB level in 3rd trimester (g/dL)	13.1 ± 1.7
Anemia	75 (7.9)
Gestational age at delivery (wk)	39.5 ± 1.1
Gestational weight gain, in kg	8.9 ± 4.5
Caesarean section	222 (23.3)
*Newborns*	
Birth weight (g)	3077 ± 406.3
Birth length (cm)	48.2 ± 1.7
Head circumference (cm)	33.1 ± 1.2
Female	457 (48)
SGA	153 (16.0)
PTB	40 (4.2)
LBW	54 (5.6)
LGA	25 (2.6)
Macrosomia	8 (0.8)

*MUAC* Mid upper arm circumference, *Hb* Haemoglobin, data are presented as mean±standard deviation or *n* (%)

**Fig. 2:**
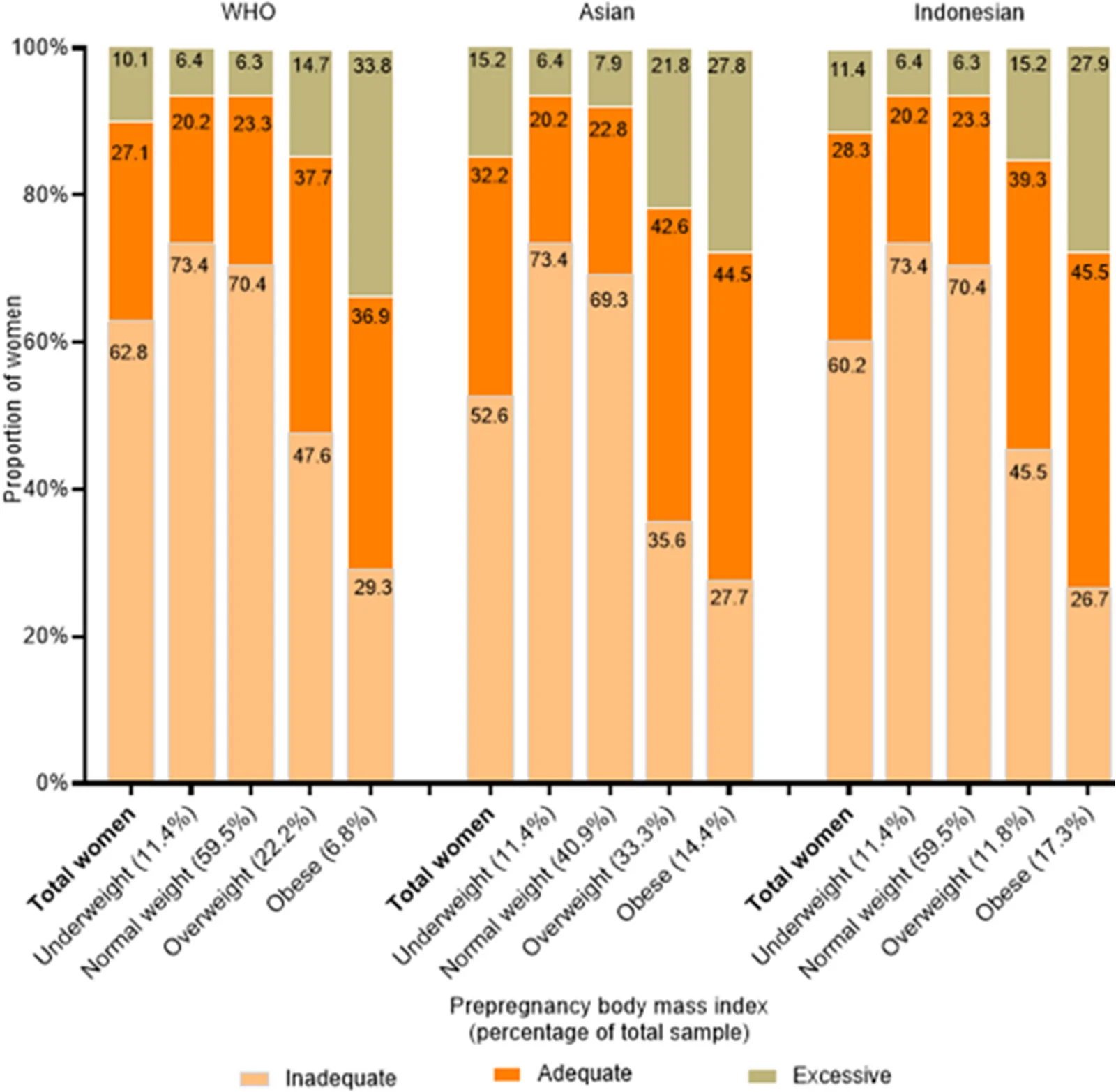
Gestational weight gain adequacy according to the IOM applied to the WHO, Asian and Indonesian pre-pregnancy body mass index cutoffs. The panel represent the proportion of women with GWG classified based on IOM and pre-pregnancy BMI (*n* = 957)

The different pre-pregnancy BMI categories according to WHO, Asian and Indonesian BMI cutoffs and associated GWG adequacy ranges when applying IOM recommendations is shown in Fig. 2. All groups had similar definitions for women with underweight (11.4%) but differed in the proportion of women with normal weight, overweight or obesity. The Asian BMI cutoffs resulted in the highest proportion of women with overweight (33.3%) compared to standard WHO and Indonesia, whereas the Indonesian BMI cutoffs had the lowest proportion of overweight (11.8%) but the highest obesity rate (17.3%) compared to WHO and Asian cutoffs. Additionally, both standard WHO and Indonesian BMI cutoffs had the same proportion of women with normal weight, which was 59.5%. Irrespective of BMI cutoffs, a considerable proportion of pregnant women who were underweight (> 70%) or had a normal weight pre-pregnancy BMI (> 69%) experienced inadequate GWG. The proportion of inadequate GWG decreased, while the proportion of adequate and excessive GWG increased, as BMI categories increased. Women categorized as overweight and obese had a higher proportion showing adequate or excessive GWG. Using the Asian BMI compared to standard WHO cutoffs shifted women from inadequate into adequate and excessive GWG categories, particularly among overweight and obese women.

The relationship between GWG adequacy and pregnancy outcomes when WHO, Asian and Indonesian BMI cutoffs were applied is depicted in Table 3. Overall findings regarding the χ² values of the associations and the outcome percentages in the GWG groups indicated that the Asian BMI cutoffs showed a stronger linear trend between GWG and SGA, the composite adverse outcome, and LGA but not for PTB, LBW, and macrosomia individually. The composite adverse outcome showed a linear trend with GWG, potentially explained by SGA which was the most prevalent adverse outcome in our participants.

**Table 3:** Distribution of GWG categories by IOM recommendations across the WHO, Asian and Indonesian BMI classifications and association with adverse pregnancy outcomes

Pregnancy outcomes	n	Gestational weight gain								
		WHO BMI cutoffs			Asian BMI cutoffs			Indonesian BMI cutoffs		
		Inadequate (n=599)	Adequate (n=258)	Excessive (n=96)	Inadequate (n=501)	Adequate (n=307)	Excessive (n=145)	Inadequate (n=574)	Adequate (n=270)	Excessive (n=109)
		n (%)	n (%)	n (%)	n (%)	n (%)	n (%)	n (%)	n (%)	n (%)
SGA	153	109 (18.2)	32 (12.4)	12 (12.5)	98 (19.6)	40 (13.0)	15 (10.3)	106 (18.5)	35 (12.8)	12 (11.3)
		**χ²=4.55**, *p* = 0.033*			**χ²=9.65**, *p* = 0.002*			**χ²=5.86**, *p* = 0.015*		
PTB	40	29 (4.8)	8 (3.1)	3 (3.1)	26 (5.2)	9 (2.9)	5 (3.4)	28 (4.9)	7 (2.6)	5 (4.7)
		χ²=1.38, *p* = 0.240			χ²=1.77, *p* = 0.183			χ²=0.62, *p* = 0.430		
LBW	54	35 (5.8)	14 (5.4)	5 (5.2)	30 (6.0)	17 (5.5)	7 (4.8)	34 (5.9)	13 (4.8)	7 (6.6)
		χ²=0.09, *p* = 0.755			χ²=0.29, *p* = 0.589			χ²=0.01, *p* = 0.922		
Any adverse outcomes	175	125 (20.9)	36 (14.0)	14 (14.6)	112 (22.4)	44 (14.3)	19 (13.1)	121 (21.1)	38 (13.9)	16 (15.1)
		**χ²=5.38**, *p* = 0.020*			**χ²=9.91**, *p* = 0.002*			**χ²=5.37**, *p* = 0.020*		
LGA	25	11 (1.8)	8 (3.1)	6 (6.3)	7 (1.4)	11 (3.6)	7 (4.8)	10 (1.7)	9 (3.3)	6 (5.7)
		**χ²=6.11**, *p* = 0.013*			**χ²=6.64**, *p* = 0.010*			**χ²=5.95**, *p* = 0.015*		
Macrosomia	8	3 (0.5)	4 (1.6)	1 (1.0)	2 (0.4)	5 (1.6)	1 (0.7)	3 (0.5)	4 (1.5)	1 (0.9)
		χ²=1.38, *p* = 0.240			χ²=0.92, *p* = 0.336			χ²=0.99, *p* = 0.319		

GWG rate was classified according to the 2009 IOM classification recommendations based on pre-pregnancy BMI. Data are presented as n (%)

*SGA* Small for gestational age, *PTB* Preterm birth, *LBW* Low birth weight

χ², *p*= Linear by linear trend association from a chi-square test

* *p* < 0.05

**Fig. 3:**
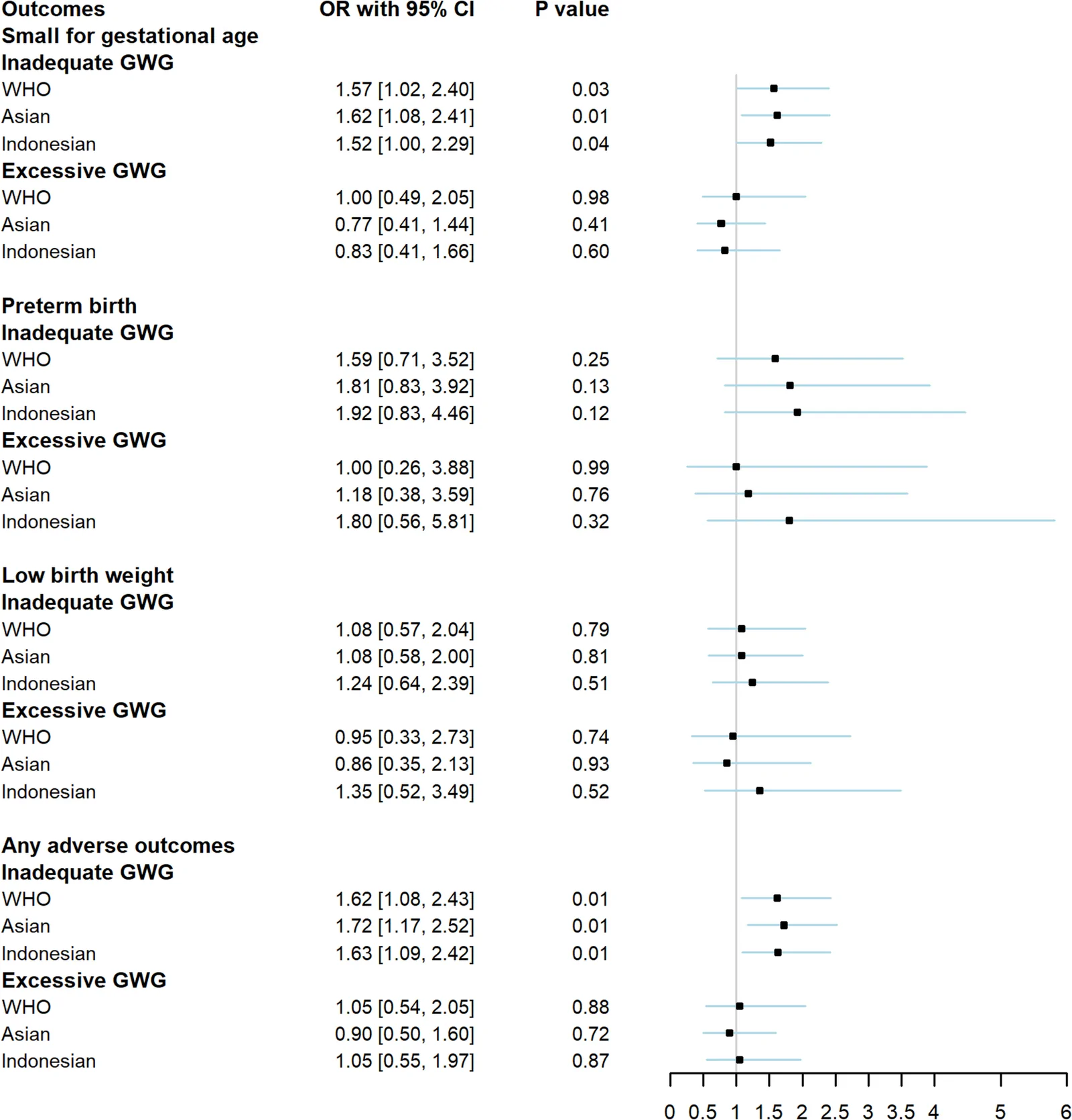
Influence of gestational weight gain categories on the risk of pregnancy outcomes when applying IOM recommendation on the WHO, Asian and Indonesian BMI cutoffs. The x-axis represents the effect size as an odds ratio (OR) of GWG adequacy and SGA, PTB, and LBW. Horizontal lines indicate the corresponding 95% confidence intervals. Reference group used in this analysis is adequate gestational weight gain. Any adverse outcomes defined as one or more of the following outcomes: SGA, PTB, LBW

The associations between GWG, categorized according to the WHO, Asian and Indonesian BMI cutoffs, and adverse pregnancy outcomes are shown in Fig. 3. Overall, the GWG classified according to Asian BMI cutoffs, showed a stronger effect size and narrower confidence interval than when using the WHO and Indonesian BMI classifications for all adverse pregnancy outcomes, yet only moderately better. Pregnancy outcomes in relation to GWG, adjusted for the total number of MMS consumed, are presented in Table 4. Inadequate GWG, regardless of BMI cutoff classification was significantly associated with an increased risk of SGA and any adverse birth outcomes. However, the Asian BMI classification showed a slightly higher and stronger association with SGA or any adverse pregnancy outcome risk compared to the WHO and Indonesian BMI classifications. Specifically, the values were 1.62 (95% CI: 1.08–2.41), 1.72 (95% CI: 1.17–2.52) for Asian, 1.57 (95% CI: 1.02–2.40), 1.62 (95% CI: 1.08–2.43) for WHO and 1.52 (95% CI: 1.00-2.29), 1.63 (95% CI: 1.09–2.42) for Indonesia BMI cutoffs, respectively. None of the BMI cutoff classifications showed any significant association between either inadequate or excessive GWG and PTB and LBW. However, using Asian BMI cutoffs we found a significant association with a reduced likelihood of LGA 0.38 (95% CI: 0.14–0.99) (*p* = 0.04), whereas the WHO and Indonesian BMI cutoffs did not show any significance. Finally, we did not find significant associations between GWG and macrosomia regardless of the BMI cutoff used (Supplementary Figure S1).

**Table 4:** Pregnancy outcomes in relation to GWG according to IOM recommendations based on the WHO, Asian and Indonesian BMI classifications

Pregnancy outcomes^b^	IOM recommendations^a^											
	WHO BMI cutoffs				Asian BMI cutoffs				Indonesian BMI cutoffs			
	Inadequate GWG		Excessive GWG		Inadequate GWG		Excessive GWG		Inadequate GWG		Excessive GWG	
	(OR 95% CI)	*p*	(OR 95% CI)	*p*	(OR 95% CI)	*p*	(OR 95% CI)	*p*	(OR 95% CI)	*p*	(OR 95% CI)	*p*
SGA	**1.54 (1.02**,** 2.39)**	0.03*	1.01 (0.49, 2.06)	0.97	**1.62 (1.08**,** 2.41)**	0.01*	0.77 (0.41, 1.45)	0.42	**1.51 (1.00**,** 2.29)**	0.04*	0.83 (0.41, 1.68)	0.61
PTB	1.56 (0.70, 3.48)	0.27	1.02 (0.26, 3.95)	0.97	1.81 (0.83, 3.92)	0.13	1.24 (0.39, 3.69)	0.73	1.91 (0.82, 4.44)	0.13	1.87 (0.57, 6.04)	0.29
LBW	1.06 (0.56, 2.01)	0.84	0.97 (0.33, 2.78)	0.95	1.08 (0.58, 2.00)	0.79	0.88 (0.35, 2.19)	0.79	1.23 (0.63, 2.38)	0.53	1.40 (0.54, 3.63)	0.48
Any adverse outcomes^c^	**1.61 (1.08**,** 2.42)**	0.02*	1.05 (0.54, 2.06)	0.86	**1.71 (1.17**,** 2.51)**	0.006*	0.90 (0.50, 1.61)	0.74	**1.62 (1.09**,** 2.41)**	0.01*	1.06 (0.56, 1.99)	0.85
LGA	0.57 (0.23, 1.45)	0.24	2.10 (0.70, 6.22)	0.18	**0.38 (0.14**,** 0.99)**	0.04*	1.37 (0.52, 3.63)	0.51	0.51 (0.20, 1.27)	0.14	1.71 (0.59, 4.94)	0.31
Macrosomia	0.32 (0.07, 1.44)	0.13	0.66 (0.07, 6.04)	0.71	0.24 (0.04, 1.25)	0.09	0.41 (0.04, 3.61)	0.42	0.35 (0.07, 1.57)	0.17	0.61 (0.06, 5.55)	0.61

^1^Reference group: Adequate GWG

^b^Adjusted for the total number of MMS consumed

^c^Composite of SGA, PTB and LBW, *p < 0.05

 We subsequently determined th e best-fitting model regarding the association between GWG and SGA using different BMI cutoffs. The AIC test score results were 797.0 for WHO, 792.2 for Asian and 796.1 for Indonesian BMI classifications. The lowest AIC score for the Asian model indicates a more favorable balance of fit and complexity compared to the WHO and Indonesian results. Additionally, the Akaike weight calculation demonstrated that the Asian model has an 80% probability of being the best model. We also conducted the AIC test for other adverse birth outcomes by additionally adjusting for the total number of MMS consumed, and these results confirmed that the Asian model remained the best choice (Supplementary Tables S1 and S2).

## Discussion

In our population-based study in Parepare district, Indonesia, most women had a normal pre-pregnancy BMI irrespective of which BMI classification was used. However, using the Asian cutoffs, we showed a larger proportion of women classified as overweight, whereas the Indonesian BMI cutoffs resulted in lower overweight but the highest obesity prevalence. Additionally, using Asian cutoffs resulted in the highest proportions of women with adequate and excessive GWG, particularly among those categorized as overweight or obese. The proportion of women with adequate and excessive GWG increased with higher BMI categories. Using the Asian BMI classification we showed a stronger linear trend with a more precise estimate of the association between GWG and SGA, as well as any adverse outcomes. In conclusion, gestational weight gain classes based on Asian BMI cutoffs corresponded better with adverse pregnancy outcomes than WHO or Indonesian BMI cutoffs.

The higher proportion of women classified as overweight and obese in the Asian BMI cutoffs compared to the standard WHO classification aligns with findings from an earlier small study conducted in Indonesia. The study reported that 21.7% of women were classified as overweight and 5.3% as obese based on Asian BMI criteria. In contrast, using WHO classifications, only 13.5% were classified as overweight and 1.1% as obese [26]. The difference can be attributed to the fact that Asian BMI cutoffs categorize individuals as overweight or obese at lower BMI values compared to the WHO cutoffs. Consequently, some women who were classified as normal or overweight under the WHO cutoffs were classified as overweight or obese under the Asian cutoffs. As the GWG adequacy ranges vary depending on pre-pregnancy BMI, this classification explains the shift to a higher proportion of women who had obesity and excessive GWG in Asian categories compared to the WHO and Indonesian classifications.

The Asian BMI classification revealed a stronger linear trend and a more robust association between GWG and adverse pregnancy outcomes compared to the WHO and Indonesian classifications. This suggests that the Asian BMI cutoffs might more accurately reflect the risk of adverse outcomes associated with GWG within this population. Previous research emphasizes the fact that inadequate GWG is associated with higher risks of SGA, and PTB [4, 27–28]. Our analysis additionally also indicated that excessive GWG increased the risk of having LGA across all BMI classifications, albeit not statistically significant. Other studies have described a significant association between excessive GWG and LGA and macrosomia [4, 28, 29]. The discrepancy with our results may be attributed to the small number of LGA cases in our study. Similarly, the prevalence of macrosomia in our population was too small to allow reliable conclusions regarding any association with GWG.

While there are variations in BMI cutoffs among Asian countries, most countries have applied lower BMI cutoffs for overweight (23-<25.0 kg/m^2^) and obesity (≥ 25.0 kg/m^2^ or ≥ 27.5 kg/m^2^) [30, 31] compared to the standard WHO cutoffs, which define overweight as 25.0–29.9 kg/m^2^ and obesity ≥ 30 kg/m^2^. The lower cutoffs are likely more sensitive in identifying the related health risks as Asians typically have a higher percentage of body fat and a greater risk of developing metabolic diseases at lower BMI levels compared to Western populations [13, 14]. Women with overweight or obesity are at a high risk of developing pre-eclampsia, GDM and complications during childbirth [6]. GDM increases the risk of cardiometabolic disease during pregnancy and post birth in the mother [32]. Moreover, babies born to women with a history of GDM also encounter an increased risk of developing obesity and other cardiometabolic disorders later in life [33]. Recent data show a high prevalence of GDM among Asian regions varying from 4.5 to 20.3% regardless of the racial/ethnic differences in BMI [34]. Moreover, excessive GWG increases the risk of gestational diabetes, preeclampsia [35, 36] and postpartum weight retention [37]. The latter can lead to long-term obesity, further raising the risk of future chronic diseases like cardiovascular disease, type II diabetes and hypertension [38]. Babies born to women who had excessive GWG are more likely to be born macrosomic [4] and develop overweight or obesity already in childhood [39]. Using lower BMI cutoffs to advise GWG in Asian women may help to monitor and prevent excessive GWG, which in turn can lead to improved maternal and infant health outcomes.

In Indonesia, different BMI cutoffs are applied for the general population to determine nutritional status and for pregnancy to determine adequate GWG. For the general population including pre-pregnant women, the cutoff for overweight and obesity is at 27.0 kg/m^2^, whereas for pregnant women the WHO cutoff of 30 is applied. As GWG adequacy is determined by pre-pregnancy BMI, implementing these two different BMI classifications within a population can be problematic and potentially lead to inaccurate health assessment, especially when determining GWG adequacy. Our analysis clearly indicated that the number of women with inadequate GWG was higher when using WHO BMI cutoffs than in the Indonesian cutoffs. Conversely, excessive GWG was more prevalent when using Indonesian cutoffs than the WHO. As a result, excessive GWG was inversely associated with the likelihood of SGA when using Indonesian cutoffs, while it showed no significant association with the risk of SGA when assessed by the WHO cutoffs. Although the analysis only indicated significance for SGA, the results indicated a similar effect for other adverse outcomes, but sample size was limited. In summary, using a single, uniform BMI cutoff, preferably the Asian classification, for pre-pregnancy and pregnant women within a population is more preferable. This would lead to several important practical and policy implications such as earlier identification of individuals at metabolic risk, improved alignment with regional evidence and enhanced comparability with Asian and global surveillance data, and support more accurate public health burden estimates for overweight/obesity, which affect program planning and resource allocation and policy implications for national health guidelines, including NCD screening protocols, community health programs, and nutrition assessment standards. In more detail, the Asian cutoffs would enhance clinical practice by increasing diagnostic sensitivity and providing more reliable prevalence estimates, ultimately improving risk stratification in primary care. This change can then facilitate earlier lifestyle counseling and guide decisions regarding metabolic screening. Additionally, these cutoffs can enhance antenatal monitoring by accurately classifying maternal overweight/obesity allowing for more appropriate antenatal counseling, and monitoring. Furthermore, the potential need to revisit National Guidelines related to non-communicable disease, nutrition, and maternal health, with potential challenges that may arise such as the need for workforce training, effective communication strategies, and adequate health system capacity, given that more individuals may be classified as overweight or obese.

### Strengths and limitations

A clear strength of the study was that prospective data collection was used to compare three different BMI classifications and to assess the association of GWG with pregnancy outcomes. However, the analysis also had limitations: (1) the pre-pregnancy weight was self-reported, which could potentially underestimate weights [40]. This, in turn, skews BMI values downward and affects BMI classification. Consequently the Indonesian, WHO and Asian classifications may underestimate true overweight and obesity when weight is underreported. However, since this misclassification is likely non-differential with respect to classification systems, it is not likely that it affects our conclusions. (2) the sample size calculation was based on the prevalence of LBW, not SGA, thus results should be seen as descriptive and exploratory; and (3) the data were collected in one district with an urban setting which limits generalization. Specifically, we note that maternal nutrition patterns, dietary diversity, adherence to supplements, healthcare access, and socioeconomic conditions vary widely across regions in Indonesia. Therefore, the associations observed in this urban setting may differ in rural, remote, or socioeconomically distinct populations. Finally, we did not collect data on the incidence of preeclampsia, hypertension, and gestational diabetes despite their known association with BMI and GWG. Further studies with a larger sample size preferably covering a multi-center setting could improve precision and confirm generalizability of our findings.

## Conclusions

We found that Asian BMI cutoffs appear to be more sensitive to identify adverse pregnancy outcomes compared to both standard WHO and Indonesian BMI classifications. These results, therefore, highlight the need to carefully review the current BMI classification used for pregnant Indonesian women. Using the most appropriate cutoffs could facilitate early and more accurate identification of at-risk women, potentially reduce pregnancy-related complications and ultimately contribute to improved maternal and neonatal health outcomes.

## Supplementary Information

Supplementary Material 1.

## Data Availability

Data used are available from the corresponding author upon reasonable request.

## Abbreviations

ANC: Antenatal care
AIC: Akaike information criteria
BMI: Body mass index
GWG: Gestational weight gain
GDM: Gestational diabetes mellitus
IOM: Institute of Medicine
LBW: Low birth weight
LGA: Large for gestational age
MCHB: Maternal child health book
MUAC: Mid upper arm circumference
MMS: Multiple micronutrient supplementation
PTB: Preterm birth
SGA: Small for gestational age
WHO: World health organization
