# Supplementary material for: Associations between gestational weight gain and pregnancy outcomes using WHO, Asian, and Indonesian BMI classifications: a prospective longitudinal study in Parepare, Indonesia
Source: BMC Public Health. 2026 Mar 14;26:3220. doi: 10.1186/s12889-026-26818-2 (PMC13737707; doi:10.1186/s12889-026-26818-2)
Supplement: Supplementary file 1 [file 12889_2026_26818_MOESM1_ESM.docx]

**Supplementary**

Supplementary file 1. Questionnaire

| **Questionnaire for baseline survey among pregnant women in Parepare,**  **South Sulawesi, Indonesia, 2021** | |
| --- | --- |
| Record ID: | Name of interviewer: |
| No ID: | Date of interview: ____/_____/_____ |
| Community Health Centre: |  |
| No mobile phone |  |
|  |  |

| **PART I. CHARACTERISTICS OF MOTHER, SOCIAL ECONOMIC STATUS AND HOME ENVIRONMENTS** | | | | | | | | | | | | |
| --- | --- | --- | --- | --- | --- | --- | --- | --- | --- | --- | --- | --- |
|  | |  | |  | | | | | | | |  |
| **S.N** | | **Questions** | | **Response categories** | | | | | | | | **Skip to** |
| 001 | | Name of responden (wife) | | **…………………………………………………** | | | | | | | |  |
| 002 | | Age of respondent | | Date …… month……. Year…….(old) | | | | | | | |  |
| 003 | | Name of husband | |  | | | | | | | |  |
| 004 | | Age of husband | | Date …… month……. Year…….(old) | | | | | | | |  |
| 005 | | Gestational age when interview done | | ………..weeks | | | | | | | |  |
| 006 | | Estimated delivery date | |  | | | | | | | |  |
| 007 | | What is the highest grade you and your husband completed?  Wife | | Completed grade________________________  Elementary school………………......................1  Junior high school………………......................2  Senior high school…………….........................3  University/ college diploma...............................4  University/college degree..................................5 | | | | | | | |  |
| 008 | | What is the highest grade you and your husband completed?  Husband | | Completed grade________________________  Elementary school………………......................1  Junior high school………………......................2  Senior high school…………….........................3  University/ college diploma...............................4  University/college degree.................................5 | | | | | | | |  |
| 009 | | What kind of work do you mostly do?  Wife | | Housewife ……………………………….........1  Daily laborer…………………………………..2  Governmental employee………………………3  Private employee………………………………4  Temporary employee..….…..………………....5  Merchant………………...…..………………...6  Farmer ………………………………...............7  Others (specify)_________________________ | | | | | | | |  |
| 010 | | What kind of work do your husband mostly do?  Husband | | Daily laborer…………………………………..1  Governmental employee………………………2  Private employee………………………………3  Merchant………………...…..………………...4 Fisherman……………………………..............5  Farmer ………………………………...............6  Temporary employee….…..………………....7  Non-employed…………………………...........8  Others (specify)_________________________ | | | | | | | |  |
| 011 | | What is your current marital status? | | Married…………………………………….......1  Divorced……………………………………….2  Widowed…………………………………........3  Other (specify)__________________________ | | | | | | | |  |
| 012 | | What is your family size? (Including you)? | | …………………………………………….. | | | | | | | |  |
| 013 | | Do you have health insurance? | | Yes………………………………………….....1  No……………………………………………..2 | | | | | | | | 015 |
| 014 | | What type of health insurance (BPJS) do you have? | | KIS (paid by government………………….......1  Mandiri (paid by herself……..………………..2  Private health insurance……………………….3  Other (specify)……………………………..….4 | | | | | | | |  |
| 015 | | What is the main source of drinking water for members of your household? | | Refill/bottled water…………………………….1  Pipe/running water …………………………….2  Tube well/borehole……………………….…....3  Protected dug well…………………………......4  Other (specify)__________________________ | | | | | | | |  |
| 016 | | What is the main source of water use for washing, bathing, cleaning, for members of your household? | | Pipe / running water ………………………….1  Tube well/borehole……………………….…...2  Protected dug well………………………….....3  Protected spring…………………………….....4  Other (specify)__________________________ | | | | | | | |  |
| 017 | | What kind of toilet facility do your household members usually use? | | Flush toilet………………………………..…...1  Squat toilet…………………………………….2  No facility /bush/field……………………........3  **Other (specify)__________________________** | | | | | | | |  |
| 018 | | Do you share this toilet facility with another household? | | 1) Yes 2) No | | | | | | | |  |
| 019 | | Which one of the following does your household own?  A mobile telephone?........................................................  A television?....................................................................  A refrigerator? ................................................................  A motorcycle ?................................................................ | | **Yes, own No, don’t own**  1…………………………………………….....2  1………………………………………….……2  1………………………………………….……2  1……………………………………….….…...2 | | | | | | | |  |
| 020 | | Whose house is the house where you are living? | | Owned……………………………………........1  Property of family…………………………......2  Rented…………………………………………3 | | | | | | | |  |
| 021 | | What is the main material of the floor? **(Record observation)** | | Earth……………………………………….......1  Wood…………………………………………...2  Cement…….…………………………………...3  Ceramic tile….………………………………...4  Other (specify)**________________________**­__ | | | | | | | |  |
| 022 | | What is the main material of the exterior walls? **(Record observation)** | | Cement ……………………………………….1  Wood………………………………………….2  Bamboo……………………………………….3  Ceramic tile….………………………………..4  Corrugated iron sheet………………………...5  Other (specify)**_______________________**­___ | | | | | | | |  |
| 023 | | What is the main material of the roof? (**Record observation**) | | Corrugated iron sheet………………………….1  Ceramic roof…………………………………..2  Bamboo………………………………………..3  Other (specify) _____________________**____** | | | | | | | |  |
| 024 | | What type of fuel does your household mainly use for cooking? | | LPG…… ……………………………………....1  Wood………………………………………......2  Other (specify)**__________________________** | | | | | | | |  |
| 025 | | Have you visited a health facility for antenatal care? | | 1)Yes 2) No | | | | | | | | 028 |
| 026 | | Which health facility did you visit for ANC? | | 1. Health centre (puskesmas) 2. Integrated service post (posyandu) 3. Supporting health centre?(pustu) 4. Other……. | | | | | | | |  |
| 027 | | How many months were you pregnant at your 1^st^ visit? | | ……………….months | | | | | | | |  |
| **PART II.MATERNAL REPRODUCTIVE CHARACTERISTICS** | | | | | | | | | | | | |
| 028 | | What was the age at which you get your first period/menstruation? | | _________________________________Years | | | | | | | |  |
| 029 | | What was the age at which you married for the first time? | | _________________________________Years | | | | | | | |  |
| 030 | | How many times have you been pregnant before this pregnancy? | | ______________________________________ | | | | | | | |  |
| 031 | | How many live births did you have before this pregnancy? | | ______________________________________ | | | | | | | |  |
| 032 | | What was your age when you gave birth to your first child? | | _________________________________Years | | | | | | | |  |
| 033 | | How long is the gap between this and the preceding birth? | | **____________Year(s)__________month(s)** | | | | | | | |  |
| 034 | | What was the mode of delivery? | | Spontaneous vaginal delivery………………....1  Cesarean section …...........................................2  Instrumental (forceps/vacuum) delivery….…...3 | | | | | | | |  |
| 035 | | Do you have any history of abortion? | | 1) Yes 2) No | | | | | | | |  |
| 036 | | Do you have any history of still birth? | | 1) Yes 2) No | | | | | | | |  |
| 037 | | Do you have any history of a caesarean section? | | 1) Yes 2) No | | | | | | | |  |
| 038 | | Have you experienced a baby born early, more than three weeks before his or her due date? | | 1) Yes 2) No | | | | | | | |  |
| 039 | | Have you experienced bleeding so much during pregnancy, birth or after giving birth that you need to be given blood? | | 1) Yes 2)No | | | | | | | |  |
| 040 | | Have you ever used modern contraceptive methods before? | | 1) Yes 2) No | | | | | | | |  |
| 041 | | Before you got pregnant, did a doctor, nurse, or other health worker tell you that you had any of the following health conditions? | | Diabetes ……………………………………….1  High blood pressure or HTN…………………..2  Mental health disorder (depression,anxiety,..)...3  None…………………………………………...4 | | | | | | | |  |
| 042 | | Did you experience any of the following complications during the index pregnancy? | | Severe nausea ……………………………........1  Severe vomiting ………………………………2  Antenatal bleeding… ……………………........3  Swollen hands/face…………………………....4  Blurred vision…………………………………5  Severe abdominal pain…….………………….6  Pain during urination…………………………7  Severe headache…….…………………...........8  Severe weakness………………………...........9  Others (specify)**_______________________________** | | | | | | | |  |
| 043 | | During this current pregnancy, have you ever had your haemoglobin levels checked | | 1. Yes 2. No | | | | | | | |  |
| 044 | | If yes, what symptoms did you experience that led you to have your haemoglobin level checked? | | Severe vomiting ………....................................1  Antenatal bleeding… ……………………........2  Swollen hands/face…………………………....3  Blurred vision…………………………………4  Severe abdominal pain…….………………….5  Severe headache…….…………………...........6  Severe weakness………………………............7  Pale skin……………………………………….8  No symptoms………………………………….9  Others specify)**__________________________** | | | | | | | |  |
|  | |  | |  | | | | | | | |  |
| **PART III. DIETARY DIVERSITY** | | | | | | | | | | | | |
| 049. **Dietary diversity:** Now I’d like to ask you everything that you **ate or** **drank yesterday during the day or night**, whether you ate it at home or anywhere else. Please try to recall all foods and drinks, any snacks or small meals, as well as any main meals and say **YES** if you ate or drank the following food groups otherwise say **NO**. (**Note for data collector:** Please ask her whether she ate or drank yesterday the following food groups with the given description and /or examples one by one and record the response). | | | | | | | | | | | | |
|  | | **Food categories** | **Descriptions and/or examples** | | | | | | | **Response** | |  |
| A | | Foods made from grains | Such as porridge, bread, rice, pasta/noodles or other foods | | | | | | | 1)Yes | 2)No |  |
| B | | White roots & tubers and plantains | Like white potatoes, cassava, or another | | | | | | | 1)Yes | 2)No |  |
| C | | Pulses | Such as mature beans or peas (fresh or dried seed), lentils or bean/pea products | | | | | | | 1)Yes | 2)No |  |
| D | | Nuts and seeds | Like any tree nut, groundnut/peanut or certain seeds, or nut/seed “butters” or pastes | | | | | | | 1)Yes | 2)No |  |
| E | | Milk and milk products | Such as milk, cheese, yoghurt or other milk products but NOT including butter | | | | | | | 1)Yes | 2)No |  |
| F | | Organ meat | Like liver, kidney, heart or other organ meats or blood-based foods | | | | | | | 1)Yes | 2)No |  |
| G | | Meat and poultry | Such as beef, lamb, goat, sheep, or chicken | | | | | | | 1)Yes | 2)No |  |
| H | | Fish and seafood | Like fresh or dried fish, shell fish or seafood | | | | | | | 1)Yes | 2)No |  |
| I | | Eggs | Eggs from poultry | | | | | | | 1)Yes | 2)No |  |
| J | | Dark green leafy vegetables | Like lettuce, water spinach, cassava leaves, cabbage, and others | | | | | | | 1)Yes | 2)No |  |
| K | | Vitamin A-rich vegetables, roots and tubers | Such as pumpkin, carrots, squash or sweet potatoes that are yellow or orange inside | | | | | | | 1)Yes | 2)No |  |
| L | | Other vegetables | Like green pepper, tomato, onion, radish, fresh corn, peas and beans as fresh pods | | | | | | | 1)Yes | 2)No |  |
| M | | Vitamin A-rich fruits | Like ripe mango and ripe papaya | | | | | | | 1)Yes | 2)No |  |
| N | | Other fruits | Such as banana, apple, avocado, lemon, orange, guava, watermelon, and cactus pear | | | | | | | 1)Yes | 2)No |  |
| O | | Condiments and seasonings | Ingredients used in small quantities for ﬂavour, such as chilies, spices, herbs, fish powder, tomato paste, ﬂavour cubes or seeds | | | | | | | 1)Yes | 2)No |  |
| P | | Other beverages and foods | Oils, fats or butter, savoury and fried snacks, sweets, sugar-sweetened beverages and other beverages and foods such as tea or coﬀee if not sweetened, clear broth, alcohol, pickles, olives and similar. | | | | | | | 1)Yes | 2)No |  |
| 050 | | What is your average meal frequency per day? | ....................times | | | | | | | | |  |
| 051 | | How frequently do you eat fruits (Daily? Weekly? Or monthly?)? How many times per the specified time? | _______times/day_______days/week_______days/month | | | | | | | | |  |
| 052 | | How frequently do you eat vegetables (Daily? Weekly? Or monthly?)? How many times per the specified time? | _______times/day_______days/week_______days/month | | | | | | | | |  |
| 053 | | How frequently do you eat foods of animal source (Daily? Weekly? Or monthly?)? How many times per that time? | _______times/day_______days/week_______days/month | | | | | | | | |  |
| 054 | | Do you drink alcohol? | 1) Yes 2) No | | | | | | | | | 056 |
| 055 | | How frequently do you drink alcohol at least 1 unit (Daily? Weekly? Or monthly?)? How many times per that time? | _______times/day_______days/week_______days/month | | | | | | | | |  |
| 056 | | Do you smoke cigarette? | 1) Yes 2) No | | | | | | | | | 058 |
| 057 | | How frequently do you smoke (Daily? Weekly? Or monthly?)? How many times per the specified time? | _______times/day_______days/week_______days/month | | | | | | | | |  |
| 58 | | Is there any family member staying in the same house smoke? | 1) Yes 2)No | | | | | | | | | 061 |
| 59 | | If Yes, How frequently do you smoke (Daily? Weekly? Or monthly?)? How many times per the specified time? | _______times/day_______days/week_______days/month | | | | | | | | |  |
| 60 | | Who are the family member smoke? | Husband………………………………………………....1  Father/father in low……………………………………...2  Brother/sister ……………………………………………3  Relatives…………………………………………………4 | | | | | | | | |  |
| 061 | | Do you drink coffee or tea? | 1) Yes 2) No | | | | | | | | | 063 |
| 062 | | How frequently do you drink coffee or tea (Daily? Weekly? Or monthly?)? How many times per the specified time? | _______times/day_______days/week_______days/month | | | | | | | | |  |
| **PART IV. PSYCHOSOCIAL HEALTH** | | | | | | | | | | | | |
| **Anxiety:** Now I would like to ask you how you have been feeling in the past one week. So, listen carefully, and tell me which comes closest to how you have been feeling. That is, tell me whether you haven’t been feeling **NOT AT ALL**, or you have been feeling **OCCASIONALLY**, **A LOT OF THE TIME** or **MOST OF THE TIME** to each of the ff. | | | | | | | | | | | | |
| **Over the past one week, ………………………………………….** | | | | Did not apply to me at all | | | Applied to me to some degree, or some of the time | | Applied to me to some degree, or some of the time | Applied to me to some degree, or some of the time | |  |
| 063 | | (s) I found it hard to wind down | | 1 | | | 2 | | 3 | 4 | |  |
| 064 | | (a) (a) I was aware of the dryness of my mouth | | 1 | | | 2 | | 3 | 4 | |  |
| 065 | | (d) I couldn’t seem to experience any positive feelings at all | | 1 | | | 2 | | 3 | 4 | |  |
| 066 | | (a) (a) I experienced breathing difficulty (e.g. excessively rapid breathing, breathlessness in the absence of physical exertion) | | 1 | | | 2 | | 3 | 4 | |  |
| 067 | | (d) I found it difficult to work up the initiative to do things | | 1 | | | 2 | | 3 | 4 | |  |
| 068 | | (s) I tended to over-react to situations | | 1 | | | 2 | | 3 | 4 | |  |
| 069 | | (a) I experienced trembling (e.g. in the hands) | | 1 | | | 2 | | 3 | 4 | |  |
| 070 | | (s) I felt that I was using a lot of nervous energy | | 1 | | | 2 | | 3 | 4 | |  |
| 071 | | (a) I was worried about situations in which I might panic and make a fool of myself | | 1 | | | 2 | | 3 | 4 | |  |
| 072 | | (d) I felt that I had nothing to look forward to | | 1 | | | 2 | | 3 | 4 | |  |
| 073 | | (s) I found myself getting agitated | | 1 | | | 2 | | 3 | 4 | |  |
| 074 | | (s) I found it difficult to relax | | 1 | | | 2 | | 3 | 4 | |  |
| 075 | | (d) I felt down-hearted and blue | | 1 | | | 2 | | 3 | 4 | |  |
| 076 | | (s) I was intolerant of anything that kept me from getting on with what I was doing | | 1 | | | 2 | | 3 | 4 | |  |
| 077 | | (a) I felt I was close to panic | | 1 | | | 2 | | 3 | 4 | |  |
| 078 | | (d) I was unable to become enthusiastic about anything | | 1 | | | 2 | | 3 | 4 | |  |
| 079 | | (d) I felt I wasn’t worth much as a person | | 1 | | | 2 | | 3 | 4 | |  |
| 080 | | (s) I felt that I was rather touchy | | 1 | | | 2 | | 3 | 4 | |  |
| 081 | | (a) I was aware of the action of my heart in the absence of physical exertion (e.g. sense of heart rate increase, heart missing a beat) | | 1 | | | 2 | | 3 | 4 | |  |
| 082 | | (a) I felt scared without any good reason | | 1 | | | 2 | | 3 | 4 | |  |
| 083 | | (d) I felt that life was meaningless | | 1 | | | 2 | | 3 | 4 | |  |
|  | | | | | | | | | | | | |
| **PART V. INTERNATIONAL PHYSICAL ACTIVITY QUESTIONNAIRE (IPAQ)** | | | | | | | | | | | | |
| Now, I would like to ask about the activities that you have done in the last 7 days. | | | | | | | | | | | | |
| *PART 1: Vigorous activities*  **Vigorous** physical activities refer to activities that take hard physical effort and make you breathe much harder than normal. Think *only* about those physical activities that you did for at least 10 minutes at a time. | | | | | | | | | | | | |
| 084 | During the **last 7 days**, on how many days did you do **vigorous** physical activities like heavy lifting, aerobics, digging, heavy construction, or climbing upstairs? | | | | | -----------Days per week  No vigorous physical activity. 086 | | | | | | |
| 085 | How much time did you usually spend doing v**igorous** physical activities on one of those days? | | | | | --------hours per day  --------minutes per day | | | | | | |
| *PART 2: Moderate activities*  Think about all the **moderate** activities that you did in the **last 7 days**. **Moderate** activities refer to activities that take moderate physical effort and make you breathe somewhat harder than normal. Think only about those physical activities that you did for at least 10 minutes at a time. | | | | | | | | | | | | |
| 086 | During the **last 7 days**, on how many days did you do **moderate** physical activities like carrying light | | | | | -------days per week  No moderate physical activity 088 | | | | | | |
| 087 | How much time did you usually spend doing **moderate** physical activities on one of those days? | | | | | ----------hours per day  ---------minutes per day | | | | | |  |
| 88 | Think about the time you spent **walking** in the **last 7 days**. This includes at work and at home, walking to travel from place to place, and any other walking that you have done solely for recreation, sport, exercise, or leisure. | | | | | | | | | | |  |
| 89 | During the **last 7 days**, on how many days did you **walk** for at least 10 minutes at a time? | | | | | -------days per week  No moderate physical activity 091 | | | | | |  |
| 90 | How much time did you usually spend **walking** on one of those days? | | | | | ----------hours per day  ---------minutes per day | | | | | |  |
| *PART 3: TIME SPENT SITTING*  The last question is about the time you spent **sitting** on weekdays during the **last 7 days**. Include time spent at work, at home, while doing course work and during leisure time. This may include time spent sitting at a desk, visiting friends, reading, or sitting or lying down to watch television. | | | | | | | | | | | | |
| 91 | During the **last 7 days**, how much time did you usually spend **sitting** on a **weekday**? | | | | | ---------hours per day  ---------minutes per day | | | | | |  |
|  | | | | | | | | | | | | |
| **PART VI. MULTIPLE MICRONUTRIENT SUPPLEMENTATIONS (MMS)** | | | | | | | | | | | | |
|  | |  | | |  | | | | | | |  |
| 92 | | How many MMS were you given? | | | ………………………………tablet | | | | | | |  |
| 93 | | How many MMS did you consume? | | | ………………………………tablet | | | | | | |  |
|  | | 1^st^ trimester | | | ………………………………tablet | | | | | | |  |
|  | | 2^st^ trimester | | | ………………………………tablet | | | | | | |  |
|  | | 3^st^ trimester | | | ………………………………tablet | | | | | | |  |
| 94 | | Where did you get the MMS | | | 1. Health centre (puskesmas) 2. Supporting health centre (pustu) 3. Integrated service post (posyandu) 4. Other……. | | | | | | |  |
| 95 | | Did you consume MMS every day? | | | 1. Yes 🡪101 2. No | | | | | | |  |
| 96 | | If no, why ? | | | 1. Forgot 2. Lazy to consume 3. Taking other vitamins 4. The colour of MMS changed 5. Running out of MMS 6. Cause a nausea 7. Others…………. | | | | | | |  |
| 97 | | Did you like the MMS? | | | 1. Yes 2. No | | | | | | |  |
| 98 | | If no, why? | | | 1. The colour is not attractive | | | | | | |  |
|  | |  | | | 2. The smells is bad | | | | | | |  |
|  | |  | | | 3. The size is big | | | | | | |  |
|  | |  | | | 4. Cause a nausea | | | | | | |  |
|  | |  | | | 5. Cause a dizzy | | | | | | |  |
|  | |  | | | 6. Cause black stools | | | | | | |  |
|  | |  | | | 7. Forgot | | | | | | |  |
|  | |  | | | 8. Diarrhea | | | | | | |  |
|  | |  | | | 9. Causes weakness | | | | | | |  |
|  | |  | | | 10. Consumed other vitamins | | | | | | |  |
| **PART VI. ANTHROPOMETRIC MEASUREMENTS** | | | | | | | | | | | | |
|  | | **Mother** | | | Frequency of measurement | | | | | | |  |
|  | |  | | | 1^st^ trimister | | | 2^nd^ trimister | | 3^rd^ trimister | |  |
| 99 | | Gestational age | | |  | | |  | |  | |  |
| 100 | | MUAC (cm) measure only once | | |  | | |  | |  | |  |
| 101 | | Weight (kg) | | |  | | |  | |  | |  |
| 102 | | Height (m) , measure only once | | |  | | |  | |  | |  |
| 103 | | Blood glucose (mmol/L) | | |  | | |  | |  | |  |
| 104 | | Cholesterol (md/dL) | | |  | | |  | |  | |  |
| 105 | | Haemoglobin level (g/dL) | | |  | | |  | |  | |  |
| 106 | | Blood pressure (mm/Hg) | | |  | | |  | |  | |  |
|  | |  | | |  | | |  | |  | |  |
|  | | **Newborn infant** | | |  | | | | | | |  |
| 107 | | Name | | | ……………… | | | | | | |  |
| 108 | | Sex | | | 1) Boy 2) Girl | | | | | | |  |
| 109 | | Date of birth | | | DD/MM/YYYY________________________ | | | | | | |  |
| 110 | | Birth weight (g) | | | …………………. | | | | | | |  |
| 111 | | Birth length (cm) | | | ……………………. | | | | | | |  |
| 112 | | Head circumference (cm) | | | ………………….. | | | | | | |  |
| 113 | | Delivery method | | | 1. Normal | | | | | | |  |
|  | |  | | | 2. Caesarian | | | | | | |  |
|  | |  | | | 3. Vacuum | | | | | | |  |
|  | | **Post-partum data** | | |  | | | | | | |  |
| 114 | | Mother survived after giving birth | | | 1. Yes 2. No | | | | | | |  |
| 115 | | Abortion/miscarriage (<20 weeks) | | | 1. Yes 2. No | | | | | | |  |
| 116 | | If the baby still birth, at what gestational age? | | | 1. 20 -27 weeks | | | | | | |  |
|  | |  | | | 2. 28 -36 weeks | | | | | | |  |
|  | |  | | | 3. >37 weeks | | | | | | |  |
| 117 | | Is the baby still alive after 7 days of birth? | | | 1. Yes 2. No | | | | | | |  |
| 118 | | Gestational age when delivery? | | | ………………………………………………. | | | | | | |  |

**Figure S1**: Influence of gestational weight gain categories based on IOM recommendations on risk of large for gestational age and macrosomia when using WHO, Asian and Indonesian BMI cutoffs. The x-axis represents the effect size as an odds ratio (OR) of GWG adequacy and large for gestational age and macrosomia. Horizontal lines indicate the corresponding 95% confidence intervals. Reference group is adequate gestational weight gain.

**Table S1**. Comparison of AIC values and Akaike weights on the association between GWG and pregnancy outcomes across different BMI cutoffs

| Pregnancy outcomes | WHO BMI cutoffs | | Asian BMI cutoffs | | | Indonesian BMI cutoffs | | |
| --- | --- | --- | --- | --- | --- | --- | --- | --- |
|  | AIC | Akaike | | AIC | Akaike | | AIC | Akaike |
|  | values | weights | | values | weights | | values | weights |
| SGA | 797.005 | 0.076 | | **792.296** | **0.805** | | 796.132 | 0.118 |
| PTB | -351.743 | 0.238 | | **-352.734** | **0.391** | | -352.625 | 0.370 |
| LBW | -78.905 | 0.312 | | **-79.102** | **0.344** | | -79.093 | 0.343 |
| Any adverse outcomes^1^ | 897.179 | 0.083 | | **892.575** | **0.833** | | 896.767 | 0.083 |
| LGA | -789.110 | 0.351 | | **-789.299** | **0.386** | | -788.537 | 0.263 |
| Macrosomia | 1853.484 | 0.285 | | **1854.551** | **0.487** | | 1853.034 | 0.228 |

^1^ Composite of SGA, PTB and LBW

**Table S2**. Comparison of AIC values and Akaike weights on the association between GWG and pregnancy outcomes across different BMI cutoffs*

| Pregnancy outcomes* | WHO BMI cutoffs | | Asian BMI cutoffs | | Indonesian BMI cutoffs | |
| --- | --- | --- | --- | --- | --- | --- |
|  | AIC | Akaike | AIC | Akaike | AIC | Akaike |
|  | values | weights | values | weights | values | weights |
| SGA | 797.878 | 0.077 | **793.183** | **0.805** | 797.033 | 0.118 |
| PTB | -354.161 | 0.232 | **-355.189** | **0.387** | -355.155 | 0.381 |
| LBW | -82.551 | 0.315 | **-82.725** | **0.343** | -82.710 | 0.341 |
| Any adverse outcomes^1^ | 897.032 | 0.083 | **892.421** | **0.833** | 896.606 | 0.083 |
| LGA | -787.792 | 0.351 | **-787.962** | **0.382** | -787.250 | 0.267 |
| Macrosomia | 1851.504 | 0.285 | **1852.578** | **0.487** | 1851.058 | 0.228 |

^*^Adjusted for the total number of MMS consumed

Lower AIC values indicate indicate better fit. Akaike weights indicate the relative likelihood of each model being the best

^1^ Composite of SGA, PTB and LBW
